# Overexpression of microRNA-211 in Functional Dyspepsia via Downregulation of the Glial Cell Line-Derived Neurotrophic Factor (GDNF) by Increasing Phosphorylation of p38 MAPK Pathway

**DOI:** 10.1155/2022/9394381

**Published:** 2022-12-14

**Authors:** Jue Wang, Sai Gu, Bo Qin

**Affiliations:** ^1^Department of Gastroenterology, The First Affiliated Hospital of Chongqing Medical University, Chongqing 400016, China; ^2^Department of Infectious Diseases, The First Affiliated Hospital of Chongqing Medical University, Chongqing 400016, China

## Abstract

**Background:**

Overexpression of miRNA-211 suppresses the differentiation of bone marrow stem cells into intestinal ganglion cells via downregulation of GDNF, a regulator of intestine barrier function. The study aimed to investigate the interaction between miR-211 and GDNF on intestinal epithelial cells.

**Methods:**

The expression levels of miR-211 and GDNF in duodenal biopsy specimens from FD patients and healthy controls were compared. Enteric glia cell (EGCs) cell line transfected with miR-211 mimics and inhibitors were used to clarify the expression levels of GDNF were analyzed by qRT-PCR and ELISA. Intestine epithelial cell (IECs) cell line cultured in medium from ECGs in different transfection conditions were used in wound healing assay, cell proliferation assay, and western blotting for evaluation of p38 MAPK phosphorylation level.

**Results:**

MiR-211 expression was significantly upregulated in the duodenal tissue of patients with FD compared to healthy subjects, whereas GDNF expression was significantly downregulated (both *p* < 0.05). Transfection with miR-211 mimics significantly decreased GDNF mRNA expression and protein secretion (*p* < 0.001). An inhibited intestinal epithelial cell wound healing (*p* < 0.05) and increased expression levels of phosphorylated p38 MAPK (*p* < 0.05) were found in IECs cultured with medium from EGCs transfected with miR-211 mimics.

**Conclusions:**

MiR-211 may downregulates GDNF mRNA and protein expression via activation of the pp38 MAPK signaling pathway. Targeting miR-211 or the MAPK pathway may be a potential intervention for FD.

## 1. Introduction

Functional dyspepsia (FD) is a chronic gastrointestinal (GI) disorder defined by upper abdominal symptoms considered to originate from the gastroduodenal region with no structural disease on routine investigation. FD is one of the most common GI disorders, with a prevalence of up to 30% [1, 2]. Studies have found GI mucosal alterations in some conditions of FD, and the changes take the form of increased permeability and are considered the result of mucosal inflammation and neural activation [2–5]. Especially, enteric glia cells (EGCs) are important for maintaining the integrity of the intestinal epithelial barrier [6, 7]. Dysregulation of the intestinal epithelial barrier is known to play a major role in the development, perpetuation, and severity of inflammatory bowel disease (IBD) [8].

*Helicobacter pylori* infection plays a pivotal role in gastric disorders, including dyspepsia and peptic ulcers [9]. The consequences of *Helicobacter pylori*-caused inflammation destroy the gastric mucous membranes, increasing the risk of gastric cancer. Some patients may develop a clonal hyperplasia of lymphocytes and lymphatic tissues, leading to gastric lymphoma [9]. TNF-*α* is known for the association with *Helicobacter pylori*-cased inflammation, and its different polymorphisms are associated with the risk of gastric cancer in different ethnic populations [10]. In addition, dyspepsia is also correlated with low intake of protein in community-dwelling adults older than 65 years [11].

Glia cell line-derived neurotrophic factor (GDNF) is secreted by EGCs and has been found to be a regulator of intestinal barrier function [12]. Studies showed that GDNF attenuated gut barrier inflammation in IBD caused by p38 MAPK-dependent phosphorylation [8, 13]. Therefore, deep investigations of the mechanisms underlying inflammation-induced intestinal epithelial barrier breakdown in IBD are needed to identify novel therapeutic approaches [12].

MicroRNAs (miRNAs) are small, noncoding RNAs that mediate posttranscriptional gene expression of the target gene. Studies have shown interactions between miRNAs and GDNF. Overexpression of miRNA-211 suppresses the differentiation of bone marrow stem cells into intestinal ganglion cells via downregulation of GDNF expression [14]. The purposes of this study were to (1) investigate miR-211 expression in duodenal biopsy specimens of patients with FD; (2) investigate the role of miR-211 on the expression of GDNF in EGCs; and (3) examine the effect of GDNF on wound healing and cell proliferation and migration in small intestine epithelial cells.

## 2. Materials and Methods

### 2.1. Participants

The FD patients included in this study were recruited from the Digestive Endoscopy Center and Gastroenterology Outpatient of Jinshan Hospital, the First Affiliated Hospital of Chongqing Medical University, between November 2017 and December 2018. The patient's chief complaint is upper abdominal dyspepsia diagnosed according to Rome IV (RC-IV) criteria. Patients were diagnosed as having FD with one or more than one symptom: (1) postprandial fullness after a meal; (2) early satiation; (3) upper middle abdominal pain; and (4) a burning sensation in the upper abdomen without any evidence of structural disease that can explain the above symptoms including gastroscopy. In addition, FD patients were subclassified as postprandial distress syndrome (PDS) and epigastric pain syndrome (EPS).

The inclusion criteria were: (1) aged 18–68 years (mainly exclude those with immature or degraded gastrointestinal tract development function to avoid affecting the analysis); (2) routine blood, urine, stool, stool occult blood, liver function, kidney function, blood sugar, ECG, and abdominal ultrasound examinations were normal; (3) electronic gastroscopy revealed no abnormalities or chronic superficial gastritis, or no organic lesions that could explain the patient's dyspeptic symptoms were found; and (4) FD symptoms appeared for at least 6 months before diagnosis and met the above diagnostic criteria in the past 3 months. Exclusion criteria: (1) gastroscopy showed organic diseases of the upper digestive tract; (2) abdominal ultrasound showed organic diseases of the digestive tract; (3) severe primary diseases in heart, liver, kidney, hematopoietic systems; (4) history of pelvic or abdominal surgery; (5) taking drugs that may affect the absorption of gastrointestinal function with the past 7 days; (6) history of drug or food allergy; (7) pregnant or breastfeeding women; (8) depressive and anxiety patients with significant mental disorders and a clear diagnosis; (9) chronic kidney disease (CKD), diabetes, chronic hepatitis, pneumonia, and other chronic diseases that may cause digestive symptoms; and (10) recent history of high-dose smoking (≥10 cigarettes per day) or high-dose drinking (≥75 g of alcohol per day).

Healthy volunteers recruited from physical examination center or health medical staffs of the same period serve as the control group of this study. Their routine blood, urine, stool, stool occult blood, liver function, kidney function, blood sugar, ECG, and abdominal ultrasound examinations were normal. Electronic gastroscopy revealed no abnormalities.

To investigate the association between miR-211 and FD, duodenal biopsy tissue specimens from 20 patients with FD and from 20 healthy controls were obtained during routine endoscopy. For PCR analysis, total RNA, including miRNAs, was extracted from biopsy specimens using the mirVana RNA Isolation Kit (Applied Biosystem, CA) according to the manufacturer's instructions. The quality of total RNA was evaluated by the Bioanalyzer RNA Nano Kit (Agilent, Santa Clara, CA), and the RNA was quantified using a Nanodrop spectrophotometer.

The project was carried out in accordance with the Declaration of Helsinki. The study was reviewed and approved by Institutional Review Board of our hospital (Approval number: 2017-457). Informed consents were obtained from all participants.

### 2.2. Cell Lines

The rat enteroglial cell line, EGC (Cat. No. CRL-2690), and the rat small intestinal epithelium cell line, IEC-6 (Cat. No. CRL-1592), were purchased from ATCC (Manassas, VA). Both were cultured in high-glucose Dulbecco's modified Eagle's medium (DMEM; Gibco, Invitrogen, CA, USA), supplemented with 10% fetal bovine serum (FBS, Gibco) at 37°C in an atmosphere of 5% CO_2_. The EGC conditioned medium (EGC-CM) were collected, centrifuged at 137 × g (1000 rpm) for 5 min, filtered through a 0.22-*μ*m syringe filter, and stored at −20°C until use. IEC-6 monolayers were cultured with or without EGC-CM as indicated.

### 2.3. Cell Transfection

The mimic control (10 nM, Cat. No. CmiR-SN0001-SN), inhibitor control (50 nM, Cat. No. CmiR-AN0001-SN), miR-211 mimic (10 nM, Cat. No. MmiR-SN0930-SN-3), miR-211 inhibitor (50 nM, Cat. No. MmiR-AN0930-SN-5) molecules were purchased from GeneCopoeia (Rockville, MD). Cells of  10^5^ cells were plated onto 6-well plates and cultured overnight. Lipofectamine 3000 Transfection Reagent (Invitrogen, Thermo Fisher Scientific, Inc.) was used to perform transfection. After 24 hours, the culture medium was replaced with serum-free DMEM, and the cells were incubated for an additional 48 hours for experimentation use.

### 2.4. qRT-PCR

The cDNA synthesis of miRNA was performed using the All-in-One First-Strand cDNA Synthesis Kit (AORT-0060; GeneCopoeia). Real-time PCR was performed using the All-in-One TM miRNA qRT-PCR Detection Kit (QP015; GeneCopoeia). The conditions for qPCR were: 42°C for 5 min, 95°C for 10 sec, 20 cycles of 95°C for 5 sec, 55°C for 30 sec, and 72°C for 30 sec. A primer sequence of cDNA was obtained from total RNA using a SMART® MMLV Reverse Transcriptase Kit (Clontech, Mountain View, CA). qPCR was performed using a FastStart Universal SYBR Green Master Kit (Sigma–Aldrich, St. Louis, MO), and all reactions were run in triplicate on a real-time PCR system (Applied Biosystems). The cycle passing threshold (*Ct*) values were recorded for GDNF, GAPDH, miR-211, and a small RNA (RNU48). MiR-211 was normalized by RNU48, and GDNF was normalized by GAPDH. The relative expressions of miR-211 and GDNF were calculated using 2^−ΔΔ*Ct*^ method. The specific primers for miR-211, RNU48, GAPDH, and GDNF were purchased from Ribobio (Shanghai, China). Primer sequences for GDNF and GAPDH/RNU48 are listed in Supplementary Table 1.

### 2.5. ELISA Assay

The GDNF level released into CM was quantified by the GDNF ELISA Kit (Cat. No. ab213901, Abcam, Cambridge, UK). The OD value of EGC-CM in 450 nm was measured.

### 2.6. Wound Healing Assay

Wound healing assays were performed using 6-well dishes. A standardized scratch was made on an IEC-6 monolayer using the tip of a 100-*µ*l pipette. The IEC-6 monolayers were cultured with EGC-CM from each group prior to wounding, and were maintained for 48 hours after wounding. The wounded areas were observed by a Motic AE31 inverted microscope (Motic China Group Co., Ltd.) and photographed at 24 and 48 hours. Wound closure was determined by the ratio of the wounded area at each time point compared with the initial scratch area using ImageJ software. The software attached to the camera marked the scale bar item when taking the photo, there is a basis for the relative length in the subsequent scientific image software analysis.

### 2.7. Cell Proliferation Assays

Cell proliferation was determined using the CCK-8 assay kit (Dojindo Laboratories, Kumamoto, Japan). In brief, WST-8 (a water-soluble tetrazolium salt) is reduced by dehydrogenases in cells to produce a yellow-colored product (formazan), which is soluble in the tissue culture medium. The amount of the formazan generated by the activity of dehydrogenases in cells is directly proportional to the number of living cells. Cells were trypsinized and replated for the CCK-8 proliferation assay at 24 and 48 hours after EGC-CM treatment. Briefly, the IEC-6 cells were seeded (5 × 10^3^ cells/well) on 6-well plates and cultured for 24 hours and 48 hours. At the indicated times (24 hours and 48 hours), 10 *μ*L of the CCK-8 solution was added to each well, cells were cultured for an additional 2 hours, and then the absorbance was measured at 450 nm using a microplate reader.

### 2.8. Western Blotting

Cultured IEC-6 cells were grown in 6-well plates; the seeding concentration and further siRNA transfection were described in the previous “Cell Transfection.” After 24 hours, the cells were subjected to Western blotting. Briefly, the IEC-6 cells were lysed using SDS lysis buffer, followed by SDS gel electrophoresis and immunoblotting. The immunoblots were treated with antibodies against phosphorylated p38 MAPK (Cat. No. #9211) and p38 MAPK (Cat. No. #9212) at a dilution of 1 : 500, and then, secondary antibodies, the respective horseradish peroxidase-labeled goat antirabbit IgG at a dilution of 1 : 3,000 in PBS. *β*-actin was used as loading control. Chemiluminescence signal detection and quantification were performed by densitometry. All antibodies were purchased from cell signaling (Danvers, MA).

### 2.9. Statistical Analysis

Continuous data are reported as mean ± standard deviation. Data of 2 groups were compared using an unpaired two-tailed Student's *t* test. When comparing MiR-211 and GDNF expression in normal subjects and FD patients, the Student's *t*-test and Mann–Whitney *U* test were used, respectively. All the statistical analyses were carried out using GraphPad Prism v6 software. A value of *p* < 0.05 is considered to indicate statistical significance.

## 3. Results

### 3.1. Expression of miR-211 and GDNF in Duodenal Tissue of Patients with FD

A total of 20 FD patients (8 males and 12 females) were included, with a mean age of 48.75 ± 10.55 (ranging from 28 to 66) years. According to the FD symptom classification criteria, 11 cases were simple PDS, 7 cases were PDS + EPS, and 2 cases were simple EPS. Among the symptoms of FD, 8 patients thought that postprandial fullness after a meal were the most obvious, 5 patients thought that early satiation were the most obvious, 4 cases were mainly belching, 2 cases were mainly epigastric pain, and 1 case was nausea.

**Figure 1:**
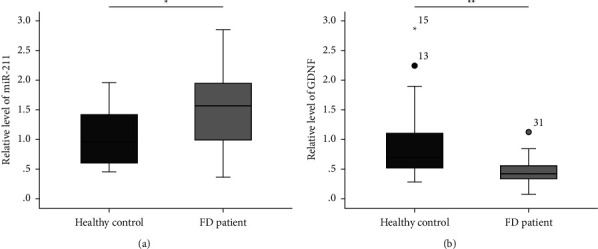
The mRNA expression level of (a) miR-211 and (b) GDNF in duodenal tissue of patients with functional dyspepsia and healthy controls (*n* = 20 for each group). FD: functional dyspepsia. ^*∗∗*^*p* < 0.05, ^*∗∗*^*p* < 0.01.

qPCR analysis indicated that miR-211 expression was upregulated in duodenal tissue of patients with FD (Figure 1(a), *p* < 0.05), whereas GDNF expression was significantly downregulated (Figure 1(b), *p* < 0.01). The raw data of QPCR results for miR-211 and GDNF expression in human duodenal biopsy tissue specimens from 20 controls and FD patients each is shown in Supplementary Table 2. Histopathological examination of duodenal biopsy specimens showed the eosinophil count (94.40 ± 75.28 vs. 64.45 ± 51.95, *p* < 0.05, Figure S1A) and mast cell count (8.95 ± 8.92 vs. 5.85 ± 4.53, *p* < 0.05, Figure S1B) in patients with FD were significantly higher than healthy controls.

### 3.2. MiR-211 Suppresses GDNF mRNA Expression in EGCs

**Figure 2:**
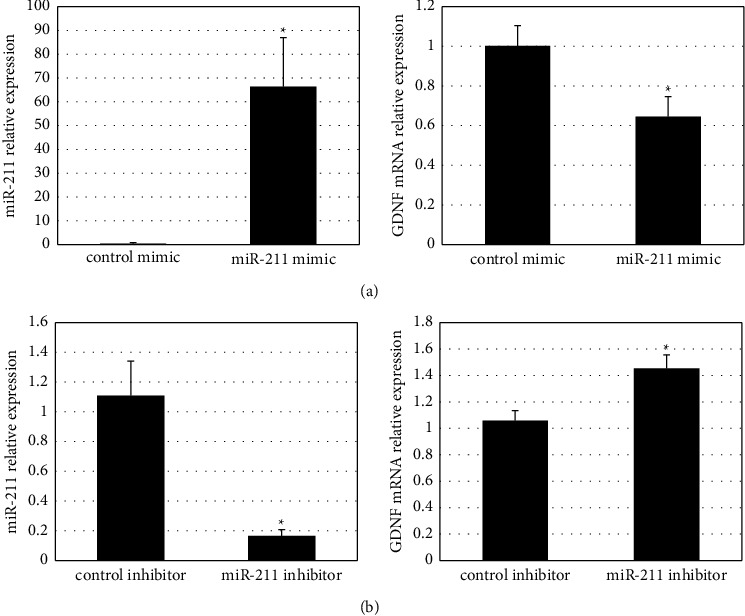
The mRNA expression level of miR-211 (left panel) and GDNF (right panel) in rat enteric glial cells. Cells are transfected with (a) miR-211 mimic, (b) miR-211 inhibitor, or the respective control. ^*∗*^*p* < 0.05.

To examine the effect of miR-211 on the transcriptional regulation of GDNF, EGCs were transfected with miRNA inhibitors or miRNA mimics, and the mRNA expressions of miR-211 and GDNF were examined at 48 hour with real-time PCR. Significantly increased miR-211 and decreased the level of GDNF mRNA expression were found in EGCs transfected with miR-211 mimic (Figure 2(a)), and vice versa in EGCs transfected with miR-211 inhibitors (Figure 2(b)), as compared with respective controls.

### 3.3. MiR-211 Suppresses GDNF Secretion

**Figure 3:**
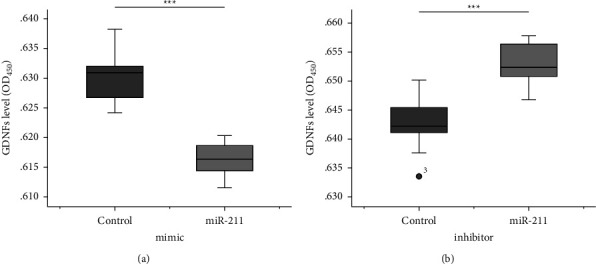
The secreted GDNF protein level in the conditioned medium from rat enteric glial cells transfected with (a) miR-211 mimic and (b) miR-211 inhibitor and the respective control. ^*∗*^*p* < 0.05, ^*∗∗*^*p* < 0.01, ^*∗∗∗*^*p* < 0.001.

Because increased GDNF mRNA expression does not directly indicate the level of secreted GDNF, GDNF levels in the culture media at 24 hours after transfection were quantified using an ELISA assay according to the manufacturer's protocol. Compared to respective controls, a significant reduction in secreted GDNF level was found in EGCs transfected with miR-211 mimic (Figure 3(a), *p* < 0.001), and vice versa in EGCs transfected with miR-211 inhibitor (Figure 3(b), *p* < 0.01).

### 3.4. Inhibition of miR-211 Increases GDNF Levels

**Figure 4:**
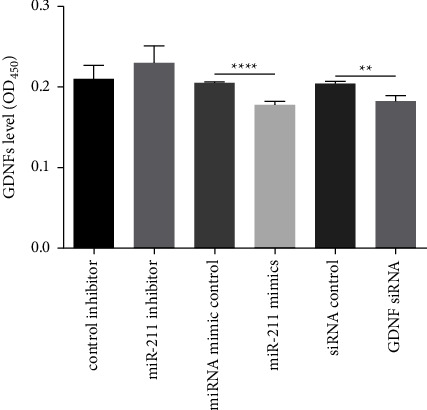
GDNF levels in the conditioned medium collected from IEC-6 cells cultured with medium from EGCs transfected with control inhibitor, miR-211 inhibitor, miRNA mimic control, miR-211 mimic, siRNA control, or GDNF siRNA, respectively. GDNF levels were determined by ELISA. EGCs: rat enteric glial cells. ^*∗∗∗*^*p* < 0.01; ^*∗∗∗∗*^*p* < 0.0001.

Small intestinal epithelium cells (IEC-6) were cultured with conditioned medium (CM) collected from the cultures of EGCs treated in 6-well plates, as described in the methods. GDNF level in the different CM was measured by ELISA (Figure 4). The level of GDNF in CM collected from EGCs transfected with miR-211 mimic was significantly lower than the control group (*p* < 0.001). The level of GDNF in CM collected from EGCs transfected with GDNF siRNA was significantly lower than that in the control group (*p* < 0.01).

### 3.5. GDNF Level Has No Effect on Wound Healing and Proliferation

**Figure 5:**
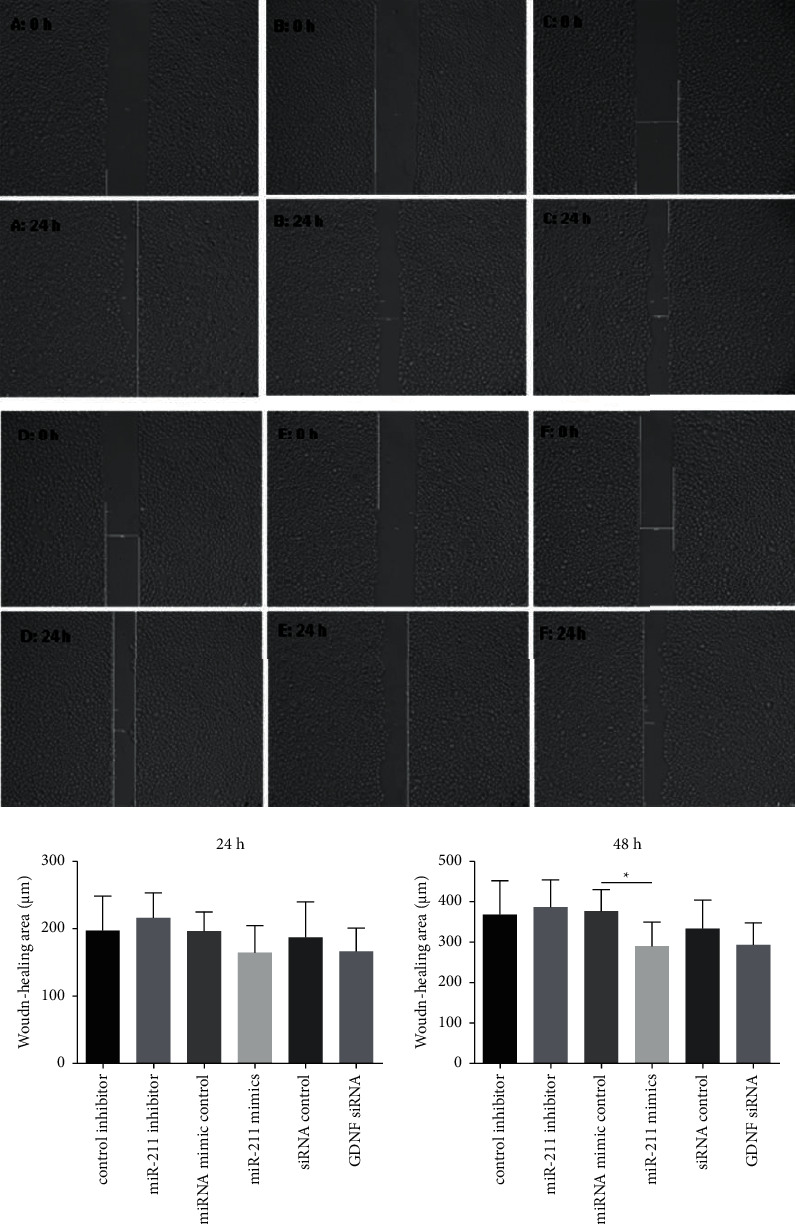
Images of scratch wounded area at time 0 and 24 hours after culture. IEC-6 monolayers were cultured with CM from (a) control inhibitor, (b) miR-211 inhibitor, (c) miRNA mimic control, (d) miR-211 mimic, (e) siRNA control, and (f) GDNF siRNA. Images were obtained with a Motic AE31 inverted microscope (magnification 50x). The left and right bar graphs illustrate the wound-healing area at 24 and 48 hours, respectively. ^*∗*^*p* < 0.05.

**Figure 6:**
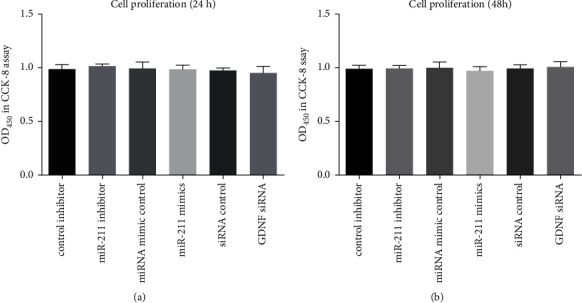
Proliferation of IEC-6 cells at (a) 24 hours and (b) 48 hours after the cells cultured with the condition medium collected from different experimental group as indicated.

Results of the wound healing assay are shown in Figure 5. No significant difference was displayed at 24 hr after transfection. At 48 hr, the level of healing in the miR-211 mimic group was significantly lower than the mimic control group (*p* < 0.05). Results of the cell proliferation assays are summarized in Figure 6. The data indicate that GDNF has no effect on cell proliferation (all *p* > 0.05).

### 3.6. GDNF Level Is Inversely Correlated with p38 MAPK Level

**Figure 7:**
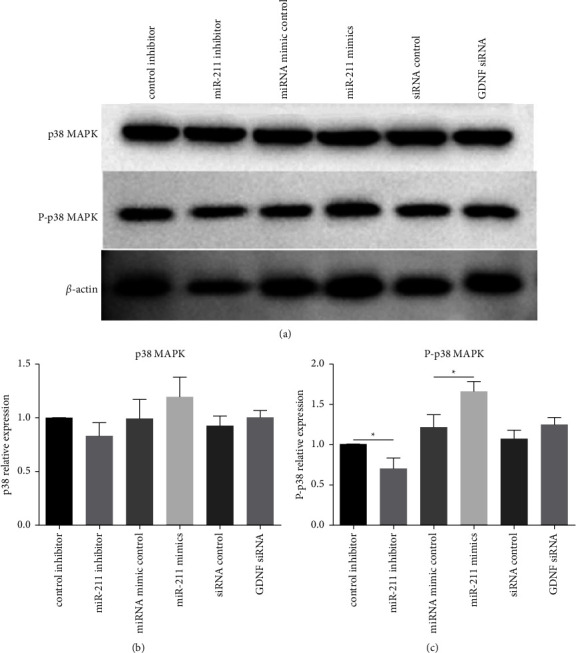
Levels of p38 MAPK and pp38 MAPK. (a) IEC-6 cells precultured with CM collected from different experimental group were lysed, and assayed for p38 MAPK and pp38 MAPK using SDS gel electrophoresis followed by immunoblotting. Chemiluminescence signal of (b) p38 MAPK and (c) pp38 MAPK was quantified by densitometry. Each assay was conducted in triplicate. ^*∗*^*p* < 0.05.

GDNF has been shown to affect p38 MAPK signaling in small intestinal epithelial cells [15]. Mechanical wounding of IEC-6 cell monolayers results in rapid activation of the extracellular signal-regulated kinase (ERK) and p38 MAP kinase pathways, and intestinal epithelial cell wound signal transduction is, at least in part, mediated by activation of the ERK and p38 MAP kinase signaling cascades [13, 15]. Therefore, we examined whether the p38 MAPK signaling pathway is activated in EGC-CM-treated IEC-6 cells. p38 MAPK were investigated in IEC-6 cells following EGC-CM application. IEC-6 cells cultured with CM collected from different experimental groups were assayed to determine p38 MAPK and pp38 MAPK levels (Figure 7). The p38 MAPK level was comparable in IEC-6 cells cultured with CM collected from the experiment groups (Figure 7(b)). The pp38 MAPK level was significantly decreased in the miR-211 inhibitor group and increased in the miR-211 mimics group as compared with respective controls (Figure 7(c), *p* < 0.05).

## 4. Discussion

Our results show that miR-211 downregulates GDNF expression through increased phosphorylation of MAPK-38 in FD, suggesting a potential of targeting miR-211 for treating FD. FD is a chronic disorder of sensation and movement in the upper digestive tract. It is well known that the enteric nervous system (ENS) plays an important role in the regulation of intestinal epithelial barrier function [16]. Moreover, GDNF is largely studied in neuromuscular pathologies, as it can prevent motor neuron degeneration in animal models of amyotrophic lateral sclerosis (ALS) and it represents a possible biomarker to predict ALS development [17]. In search of new therapeutic agents for neurodegenerative disorders, special interest has been devoted to neurotrophins because of their potential to promote survival and neurotics' growth as well as influence the differentiation of several neuronal populations. GDNF has received lots of attention because it has been shown to be a potent survival factor for dopaminergic midbrain and spinal cord neurons [17–19]. They identified astrocytes as a possible endogenous source of GDNF and suggested that astrocyte-derived GDNF can protect from neurodegeneration through inhibition of neuroinflammation.

Therapeutic inhibition of miRNAs using antisense oligomers (called anti-miRs) has also been shown to reduce tumor growth [20]. Zhang et al. reported that miRNAs mediate GDNF-induced proliferation and migration of glioma cells [21]. Recent studies revealed a strong connection between the regulatory mechanism of miRNA and disease etiology [22, 23]. A number of miRNAs have been associated with different intestinal disorders, and targeting miRNAs is a promising method of treatment for gastrointestinal disorders [24].

The main mediator secreted by enteric glial cells is the GDNF, which has effects on proliferation on glial cells and neurons in the embryonic development of the enteric nervous system [25]. Moreover, it has been consistently reported that reduced levels of GDNF lead to morphological and functional abnormalities of intestinal barrier function in mice similar to IBD [26]. EGCs can be classified into 4 main types (I–IV) according to their shapes and locations in the gut wall, but the precise role of each type of EGCs in gastrointestinal physiology and pathophysiology is not well understood [13, 27]. EGCs have been traditionally considered as a mechanical support for enteric neurons, and they are able to release a wide range of factors responsible for the development, survival, and differentiation of peripheral neurons [13, 27, 28]. Nowadays, besides their supportive role, it has been demonstrated that EGCs possess a more complex nature, playing a leading role in the maintenance of intestinal homeostasis [28]. The results of this study demonstrated that miR-211 expression is inversely related with GDNF mRNA expression and protein levels in EGCs. This association was also demonstrated in duodenal biopsy tissue specimens of patients with FD. These results suggest that miR-211 may play a role in the pathogenesis of FD by regulating GDNF.

In the present study, GDNF level was positively correlated with the wound-healing capacity of intestinal epithelium cells (IEC-6 cells) but has no effect on cell proliferation. While the length of time of treatment can affect results, the treatment time of 48 hours used in this study should have been sufficient as it is similar to the time used in other studies [29]. We cannot exclude the possibility that GDNF is inactivated during the process of collecting the CM. It is also possible that other factors in the unpurified medium may also adversely affect the activity of GDNF.

Accumulating evidence shows that many gastrointestinal disorders are associated with intestinal permeability defects, epithelial barrier dysfunction, and inflammation [3, 30, 31]. GDNF is an important regulator of intestinal barrier function [25], and studies have shown that GDNF reduces inflammation and promotes wound healing in intestinal epithelial cells [15, 32, 33]. The results of the current study are consistent with those of prior studies, which indicate that GDNF has a protective/healing role with respect to intestinal epithelial cells.

GDNF predominantly binds to GFR*α*1, NRTN to GFR*α*2, ARTN to GFR*α*3, and PSPN to GFR*α*4. Various signaling pathways are activated, such as RAS/mitogen activated protein kinase (MAPK), RAS/extracellular signal-regulated kinase (ERK), phospholipase C gamma (PLC*γ*), phosphatidylinositol-3-kinase (PI3K)/protein kinase B (AKT), and c-Jun N-terminal kinase (JNK) pathways, which contribute to cell adhesion, migration, proliferation, differentiation, and survival. Recent studies indicate that GDNF also derives from smooth muscle cells and enterocytes in significant amounts [34]. Loss of GDNF in experimental models leads to morphological and functional abnormalities in patients with IBD [33]. Although little is known about the role of p38 MAPK signaling in the regulation of intestinal barrier properties, phosphorylation of p38 MAPK was shown to activate the myosin-light chain kinase [35]. This leads to contraction of the actin-myosin ring to which the junctional complex is tethered via adaptor proteins and thereby induces destabilization of the epithelial barrier [34]. In line with these observations, the application of SB-202190 on immature Caco2 cells to mimic the inhibition of p38 MAPK signaling led to epithelial barrier stabilization. Activation of p38 MAPK signaling using anisomycin led to increased permeability and blocked GDNF-induced barrier stabilization [12]. Therefore, it can be concluded that GDNF-induced inhibition of p38 MAPK signaling contributes to barrier maturation. The predominant role of p38 MAPK signaling in the context of intestinal epithelial barrier regulation is also supported by previous study in which TNF-*α*-induced loss of intestinal barrier integrity was mediated by activation of p38 MAPK [12]. In a second step, we investigated the effects of GDNF on p38 MAPK signaling. Intestinal epithelial wound signal transduction is, at least in part, mediated by activation of p38 MAP kinase signaling cascades [12]. Our results showed that GDNF level is inversely correlated with pp38 MAPK. It suggests that GDNF may improve wound healing via pp38 MAPK suppression.

GDNF also has immunomodulatory effects, and GDNF has been shown to significantly reduce inflammation in a study using a model of chronic inflammatory bowel disease [36]. It has been suggested that the effect is due to GDNF blocking the secretion of various proinflammatory cytokines, such as TNF-*α* and interleukin (IL)-1*β* [19]. We also found that both eosinophils and mast cells increased significantly in numbers in FD patients, which is consistent with previous studies [37]. In the study conducted by Tanaka et al., GDNF level is higher in FD patients as compared to controls [37]. However, our study demonstrated a significant decrease in GDNF level in FD patients. Such discrepancy may be due to differences in the sample size, where our study had a sample size of 26 patients and 26 controls, while it was 5 patients and 9 controls of Tanaka's. Indeed, we have found some FD patients are with noticeable higher levels of GDNF, but the mean value of GDNF of all patients is reduced. We suggested that other factors except miR-211 may play a role in the regulation of GDNF, but further studies are required to evaluate this hypothesis. And how miRNAs regulate the expression of GDNF in the nervous system has signs to follow. In the Parkinson diseases (PD) model, GDNF specifically increases the expression of miR-182-5p and miR-183-5p in primary midbrain neurons [35]. Moreover, miR-9, miR-96, miR-133, and miR-146a negatively regulate GDNF level during kidney development [18]. Interestingly, Xia et al. describe miR-211 was upregulated in ganglion cell dysplasia. Whether GDNF expression was decreased in these patients needs further elucidation [14].

## 5. Conclusions

MiR-211 may downregulate GDNF via upregulation of the p38 MAPK signaling pathway, suggesting a potential intervention for FD. Studying the relationship between miR-211 and p38 MAPK is the future plan.

## Data Availability

The data used to support the findings of this study are available from the corresponding author upon request.

## Supplementary Materials

Supplementary Materials
*Supplementary Table 1*: PCR primers for GAPDH and GDNF. *Supplementary Table 2*: raw data of QPCR results of miR-211 and GDNF of human duodenal biopsy tissue specimens from control and FD groups (*n* = 20 for each). *Supplementary Figure S1*: eosinophil and mast cell counts in duodenal tissue specimens from patients with functional dyspepsia and healthy controls.
