# Supplementary material for: Overexpression of microRNA-211 in Functional Dyspepsia via Downregulation of the Glial Cell Line-Derived Neurotrophic Factor (GDNF) by Increasing Phosphorylation of p38 MAPK Pathway
Source: Can J Gastroenterol Hepatol. 2022 Dec 14;2022:9394381. doi: 10.1155/2022/9394381 (PMC9771656; doi:10.1155/2022/9394381)

Supplementary Table 1. PCR primers for GAPDH and GDNF.

| Primer | Forward | Reverse |
| --- | --- | --- |
| GAPDH | 5’-acggcaagttcaacggcacagtca-3’ | 5’-ccacgacatactcagcaccagcatca-3’ |
| GDNF | 5’-atgtcgtggctgtctgcctggtgtt-3’ | 5’-ttcgaggaagtgccgccgcttgtt-3’ |
| RNU48 | 5’-agtgatgatgaccccaggtaa-3’ | 5’-gtgatggcatcagcgacaca-3’ |

All primers were purchased from Origene, Rockville, MD.

**Supplementary Table 2.** Raw data of QPCR results of miR-211 and GDNF of human duodenal biopsy tissue specimens from control and FD groups (n=20 for each).

| \| miR-211 \|  \|  \| GDNF \|  \| \| --- \| --- \| --- \| --- \| --- \| \| Ctrl \| FD \|  \| Ctrl \| FD \| \| 1.4142 \| 1.2142 \|  \| 1.0158 \| 0.3727 \| \| 1.6935 \| 0.79 \|  \| 0.8033 \| 0.5491 \| \| 0.5864 \| 2.0705 \|  \| 0.6358 \| 0.5687 \| \| 0.473 \| 0.361 \|  \| 0.5683 \| 0.3054 \| \| 0.5396 \| 0.895 \|  \| 1.1991 \| 0.2734 \| \| 1.3104 \| 1.4439 \|  \| 0.6696 \| 0.8403 \| \| 0.5987 \| 1.9588 \|  \| 0.6589 \| 0.5424 \| \| 0.933 \| 1.8532 \|  \| 0.638 \| 0.3561 \| \| 1.6133 \| 1.0867 \|  \| 0.3604 \| 0.4853 \| \| 0.4506 \| 1.9319 \|  \| 0.3989 \| 0.5645 \| \| 0.7684 \| 2.8481 \|  \| 0.7412 \| 1.1221 \| \| 1.9588 \| 1.1567 \|  \| 0.4717 \| 0.4172 \| \| 1.4241 \| 2.1585 \|  \| 2.2473 \| 0.4289 \| \| 0.6462 \| 2.2501 \|  \| 1.7181 \| 0.3759 \| \| 0.5743 \| 0.6507 \|  \| 2.8747 \| 0.5042 \| \| 1.4743 \| 1.6702 \|  \| 0.2862 \| 0.2079 \| \| 0.8123 \| 1.4641 \|  \| 0.7074 \| 0.0786 \| \| 1.1096 \| 0.7684 \|  \| 0.8034 \| 0.1859 \| \| 0.9727 \| 1.879 \|  \| 1.8974 \| 0.3937 \| \| 1.257 \| 1.7291 \|  \| 0.3175 \| 0.8154 \| |  |
| --- | --- | --- | --- | --- | --- | --- | --- | --- | --- | --- | --- | --- | --- | --- | --- | --- | --- | --- | --- | --- | --- | --- | --- | --- | --- | --- | --- | --- | --- | --- | --- | --- | --- | --- | --- | --- | --- | --- | --- | --- | --- | --- | --- | --- | --- | --- | --- | --- | --- | --- | --- | --- | --- | --- | --- | --- | --- | --- | --- | --- | --- | --- | --- | --- | --- | --- | --- | --- | --- | --- | --- | --- | --- | --- | --- | --- | --- | --- | --- | --- | --- | --- | --- | --- | --- | --- | --- | --- | --- | --- | --- | --- | --- | --- | --- | --- | --- | --- | --- | --- | --- | --- | --- | --- | --- | --- | --- | --- | --- | --- | --- |

**Supplementary Figure 1.** Eosinophil and mast cell counts in duodenal tissue specimens from patients with functional dyspepsia and healthy controls.


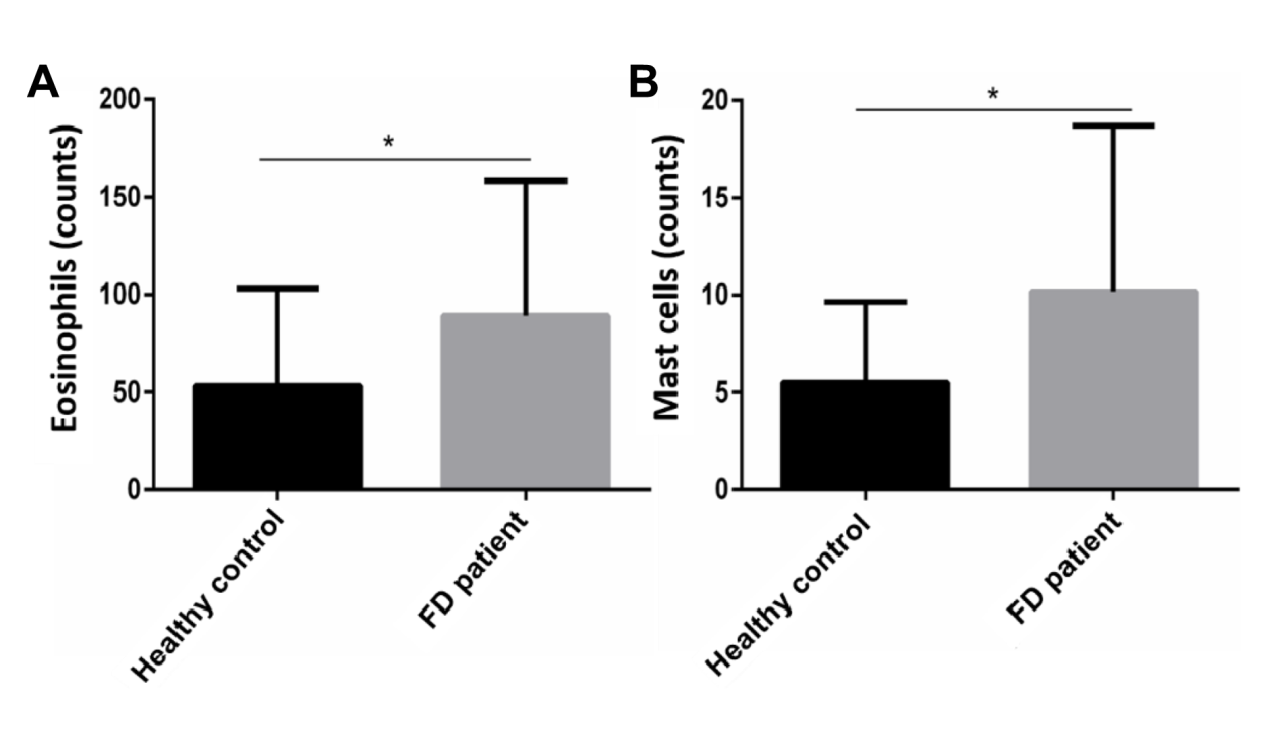

Supplement: Supplementary Materials — Supplementary Table 1: PCR primers for GAPDH and GDNF. Supplementary Table 2: raw data of QPCR results of miR-211 and GDNF of human duodenal biopsy tissue specimens from control and FD groups (n = 20 for each). Supplementary Figure S1: eosinophil and mast cell counts in duodenal tissue specimens from patients with functional dyspepsia and healthy controls. [file 9394381.f1.docx]
